# D- and L-lactate dehydrogenases during invertebrate evolution

**DOI:** 10.1186/1471-2148-8-268

**Published:** 2008-10-01

**Authors:** Melania E Cristescu, David J Innes, Jonathon H Stillman, Teresa J Crease

**Affiliations:** 1 University of Windsor, Great Lakes Institute for Environmental Research, 401 Sunset Avenue, Windsor, Ontario, N9B 3P4, Canada; 2 Department of Biology, Memorial University of Newfoundland, St. John's, Newfoundland, A1B 3X9, Canada; 3 San Francisco State University, Romberg Tiburon Center for Environmental Studies, 3152 Paradise Drive, Tiburon, CA 94920, USA; 4 University of Guelph, Department of Integrative Biology, 488 Gordon Street, Guelph, Ontario, N1G 2W1, Canada

## Abstract

**Background:**

The L-lactate and D-lactate dehydrogenases, which are involved in the reduction of pyruvate to L(-)-lactate and D(+)-lactate, belong to evolutionarily unrelated enzyme families. The genes encoding L-LDH have been used as a model for gene duplication due to the multiple paralogs found in eubacteria, archaebacteria, and eukaryotes. Phylogenetic studies have suggested that several gene duplication events led to the main isozymes of this gene family in chordates, but little is known about the evolution of *L-Ldh *in invertebrates. While most invertebrates preferentially oxidize L-lactic acid, several species of mollusks, a few arthropods and polychaetes were found to have exclusively D-LDH enzymatic activity. Therefore, it has been suggested that L-LDH and D-LDH are mutually exclusive. However, recent characterization of putative mammalian D-LDH with significant similarity to yeast proteins showing D-LDH activity suggests that at least mammals have the two naturally occurring forms of LDH specific to L- and D-lactate. This study describes the phylogenetic relationships of invertebrate L-LDH and D-LDH with special emphasis on crustaceans, and discusses gene duplication events during the evolution of *L-Ldh*.

**Results:**

Our phylogenetic analyses of L-LDH in vertebrates are consistent with the general view that the main isozymes (LDH-A, LDH-B and LDH-C) evolved through a series of gene duplications after the vertebrates diverged from tunicates. We report several gene duplication events in the crustacean, *Daphnia pulex*, and the leech, *Helobdella robusta*. Several amino acid sequences with strong similarity to putative mammalian D-LDH and to yeast DLD1 with D-LDH activity were found in both vertebrates and invertebrates.

**Conclusion:**

The presence of both *L-Ldh *and *D-Ldh *genes in several chordates and invertebrates suggests that the two enzymatic forms are not necessarily mutually exclusive. Although, the evolution of *L-Ldh *has been punctuated by multiple events of gene duplication in both vertebrates and invertebrates, a shared evolutionary history of this gene in the two groups is apparent. Moreover, the high degree of sequence similarity among D-LDH amino acid sequences suggests that they share a common evolutionary history.

## Background

The reduction of pyruvate to L(-)-lactate and D(+)-lactate is catalyzed by different NAD-dependent enzymes, the L-lactate (L-LDH: L-lactate:NAD^+ ^oxidoreductase, EC 1.1.1.27) and D-lactate dehydrogenases (D-LDH: D-lactate:NAD^+ ^oxidoreductase, EC 1.1.1.28) as well as by NAD-independent (cytochrome) enzymes (DLD: D-lactate ferricytochrome c oxidoreductase, EC 1.1.2.4). Despite their apparent functional similarity, these classes of enzymes are selective for the D/L chirality of the substrate [1]. Studies on the primary amino acid structures of L-LDH and D-LDH suggest that the genes encoding them are not evolutionarily related [2,3] and that their products belong to larger families of enzymes: L(-)-LDHs belong to the L-specific NAD-dependent dehydrogenases, while D(+)-LDHs belong to the D-isomer specific 2-hydroxy acid dehydrogenases and the FAD-binding oxidoreductase/transferase type 4 family.

L-LDH has been among the most studied enzyme families, but very little is known about the structure, function, and kinetics of D-LDH [4,5]. The main question in the evolution of L-LDH relates to the orthology of the gene loci encoding the proteins with various enzyme activities [6,7]. While L-LDHs have been extensively studied in vertebrates, there is much less information on these enzymes in invertebrates. With the availability of new *L-Ldh *sequences from two crustaceans (*Daphnia pulex *in the Branchiopoda, and a second malacostracan, the porcelain crab, *Petrolisthes cinctipes *in the Decapoda) and a few other invertebrates such as the leech *Helobdella robusta*, the polychaete *Capitella capitata*, and the tunicate *Ciona intestinalis*, we sought to gain a deeper understanding of the relationship between invertebrate and protochordate L-LDHs and those of vertebrates, and to elucidate the evolutionary relationship among invertebrate *L-Ldh*s. Moreover, the recent description of mammalian D-LDH enzymes that show significant similarity to yeast proteins with D-LDH activity [5] prompted our search for sequences with putative D-LDH activity in both vertebrate and invertebrate genomes.

## Results

### Alignments and phylogenetic analyses of L-LDH amino acid sequences

The L-LDH alignment used in phylogenetic analyses includes 315 amino acids, with 68 constant characters and 214 parsimony-informative characters. Maximum-parsimony (MP) analysis of 49 L-LDH sequences using the tree-bisection-reconnection (TBR) algorithm found two most parsimonious trees of 1982 steps long with a consistency index (CI) = 0.48, a homoplasy index (HI) = 0.51 and a retention index (RI) = 0.63. The uncorrected number of amino acid differences per site between invertebrate and chordate groups is 0.38 ± 0.019.

**Figure 1:**
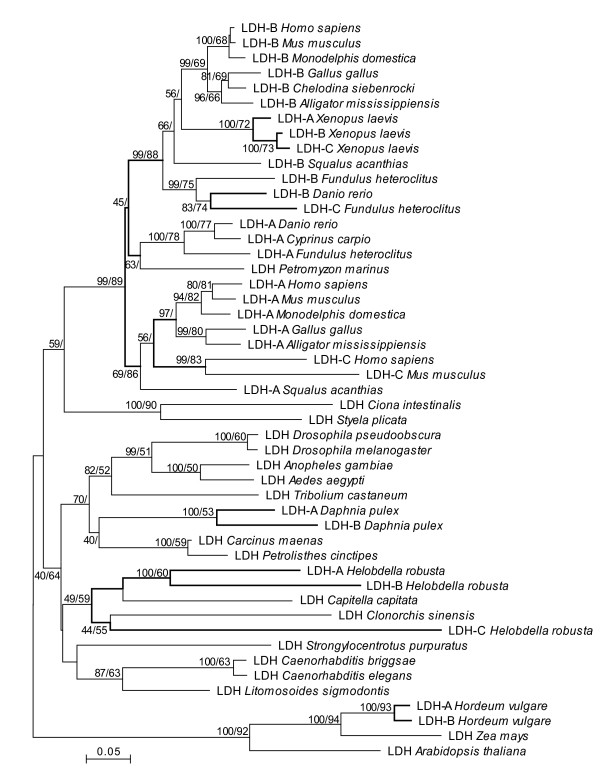
**Neighbor-joining tree based on 49 L-Lactate dehydrogenase amino acid sequences from 31 taxa.** Numbers at nodes indicate the Neighbour-joining and Maximum Parsimony percentage bootstrap support with 2,000 and 100 replicates, respectively. Nodes supported only by the Neighbor-Joining analysis show a single bootstrap value. The scale bar indicates levels of amino acid sequence divergence. The tree was rooted using the plant LDH sequences.

**Figure 2:**
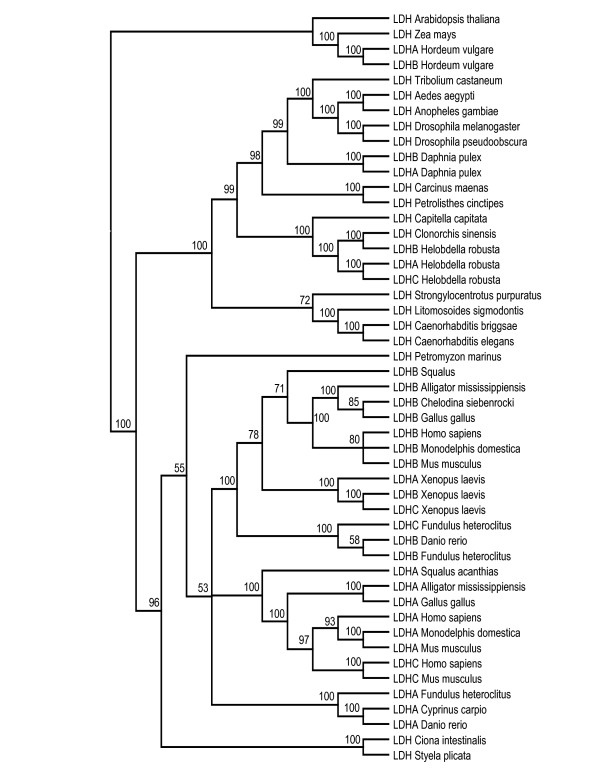
**Bayesian inference, 50% majority rule consensus tree based on 49 L-Lactate dehydrogenase amino acid sequences.** The numbers at the nodes are posterior probabilities expressed as percentages.

Phylogenetic trees generated by MP and Neighbor-joining (NJ, Figure 1) and Bayesian Inference (BI, Figure 2) all support a deuterostome cluster. The only exception is the echinoderm, *Strongylocentrotus purpuratus*, which groups with nematodes, although bootstrap support for this phylogenetic relationship is extremely low (Figure 1). The vertebrate sequences form a well-supported cluster with the LDH-A and LDH-B isozymes separating into distinct groups. Even so, there are examples of species whose A, B and C isozymes cluster with one another (e.g. *Xenopus laevis*).

The arthropod LDH sequences form a well-resolved cluster, as do the insects within it. Two copies of *Ldh *were found in the *Daphnia pulex *genome, and three copies in the *Helobdella robusta *genome. As with other cases of gene duplication outside chordates, the suffixes A, B and C do not denote orthology to the vertebrate A, B and C isozymes. The predicted protein sequences from the two *Daphnia *paralogs show 0.17 ± 0.012 amino acid divergence (p-distance) and 0.224 ± 0.014 nucleotide divergence (p-distance) in the coding regions. They cluster with one another indicating that this gene duplication occurred after the divergence of *Daphnia *from the other crustaceans, the decapods *Carcinus maenas *and *Petrolisthes cinctipes *(Figure 1 and 2). The insects and crustaceans are reciprocally monophyletic in the MP and NJ trees (Figure 1), although bootstrap support for the crustacean node is very low. Conversely, very strong support for an arthropod clade in which the crustaceans are paraphyletic relative to the insects was obtained in the BI tree (Figure 2).

The level of amino acid divergence between the three *Helobdella robusta *LDH proteins (0.32 ± 0.026, 0.46 ± 0.028, 0.52 ± 0.028) is higher than that between the *Daphnia *copies or *Fundulus heteroclitus *(LDH-B and LDH-C, 0.21 ± 0.028) and much higher than that between the recently diverged *Xenopus laevis *copies (0.019 ± 0.008, 0.049 ± 0.013, 0.063 ± 0.014). Moreover, the relationships among the three genes differs among the phylogenetic trees. Only two of the three *H. robusta *genes (*Ldh-A *and *Ldh-B*) are clearly paralogous in the MP/NJ tree, and duplicated after the divergence of *H. robusta *from the other annelid in the analysis (the polychaete, *Capitella capitata*). However, the annelid cluster also contains the trematode flatworm, *Clonorchis sinensis*, although bootstrap support for these relationships is low (Figure 1). The annelid/trematode clade is also recovered in the BI tree (Figure 2), although in this case, *C. capitata *is the sister group to the three leech and the flatworm sequences.

### Alignments and phylogenetic analyses of D-LDH amino acid sequences

The D-LDH alignment included 486 amino acids with 73 conserved sites and 319 parsimony informative sites. The uncorrected number of amino acid differences per site averaged over all sequence pairs between chordates and invertebrates is 0.41 ± 0.015.

**Figure 3:**
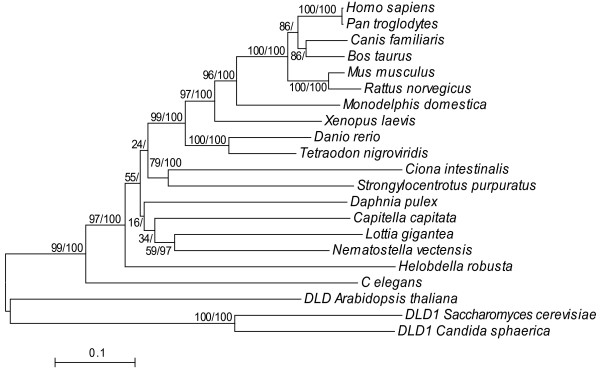
**Neighbor-joining tree based on D-Lactate dehydrogenase amino acid sequences from 21 taxa.** Numbers at nodes indicate the percentage of NJ bootstrap analyses with 2,000 replicates and posterior probabilities expressed as percentages. The scale bar indicates levels of amino acid sequence divergence. The tree was rooted using the fungal DLD1 proteins.

In general, the topology of the NJ tree generated from these sequences (Figure 3) shows that the deuterostomes form a distinct cluster relative to the other animals, as expected. For example, there is strong bootstrap support for a vertebrate cluster, and the non-vertebrate deuterostomes (*C. intestinalis *and *S. purpuratus*) cluster with them, although unexpectedly, *C. intestinalis *(a tunicate) clusters with *S. purpuratus *(an echinoderm) instead of the vertebrates. Relationships among the other invertebrates are not well resolved. For example, the annelids, *H. robusta *and *C. capitata*, do not cluster with each other, and the protostomes themselves do not form a monophyletic group (*H. robusta *is the sister taxon to all the other animals except *C. elegans*), but bootstrap support for several of the invertebrate nodes is low. Overall, this tree strongly suggests that *D-Ldh *was present in the common ancestor of animals.

## Discussion

### L-lactate dehydrogenases in vertebrate and invertebrate evolution

The majority of taxa of jawed vertebrates contain three isozymes of L-LDH (LDH-A, LDH-B and LDH-C) encoded by three loci. The M form (LDH-A) is found predominantly in white skeletal muscle (fast twitch glycolytic fibers) and is best suited for pyruvate reduction in anaerobic conditions, while the H form (LDH-B) is found in more aerobic tissues such as heart and brain and is most efficient for lactate oxidation. The X form (LDH-C) is found in various tissues such as the spermatozoa of mammals and birds, the eye lenses of birds and crocodilian and the liver and eye of teleosts [8,9].

It is commonly accepted that new metabolic capacities of L-LDH enzymes have often arisen by gene duplications in addition to more orthodox evolutionary changes in existing genes. For this reason the *Ldh *gene family has been used as a model for gene duplication in vertebrate evolution [8,10]. It is generally accepted that the *Ldh *genes of jawed vertebrates arose as a series of gene duplications in early vertebrate evolution, after the divergence of vertebrates from tunicates. However, the succession of these gene duplication events is not well understood [9,11,12]. Two main evolutionary scenarios have been proposed. The classical scenario involves the duplication of an *Ldh-A*-like locus in Agnatha (lampreys have a single LDH form) that gave rise to *Ldh-A *and *Ldh-B*. A second round of gene duplication involved *Ldh-B *and gave rise to *Ldh-B *and *Ldh-C*. Several phylogenetic studies support this hypothesis of an original *Ldh-A *and *Ldh-B *gene duplication followed by more recent and independent origins of the *Ldh-C *genes in teleost fish, *Xenopus laevis*, pigeon and mammals [7,10,13]. A second school of thought suggests that the primordial vertebrate LDH was an LDH-C like enzyme. This hypothesis emerged from phylogenetic reconstructions and from the observation that the single LDH isozyme of the primitive agnathan, the sea lamprey, is immunologically more similar to LDH-C in teleost fish than to LDH-A or LDH-B [14-16].

Our phylogenetic analyses of L-LDH isozymes are consistent with the general view of a major gene duplication event near the origin of vertebrates. Both NJ and MP phylogenies suggest that the vertebrate isozymes LDH-A and LDH-B evolved through a series of gene duplications soon after the vertebrates diverged from tunicates. The main difference between the MP, NJ and BI reconstructions is that MP and BI supports a sister group relationship between LDH in *Petromyson marinus*, the only chordate with a single LDH locus, and all of the other vertebrate LDHs. Conversely, this sequence clusters with fish LDH-A in the NJ tree. Although the controversial phylogenetic position of the LDH-C isozymes cannot be easily settled, it is clear from our results that *Ldh-C *in mammals, *X. laevis *and *Fundulus *have independent, derived origins from either *Ldh-A *or *Ldh-B*-like ancestors.

The relationship of invertebrate and protochordate *Ldh *to vertebrate *Ldh *is not well understood. Although most invertebrates seem to possess one copy of *L-Ldh *[17-19], several events of gene duplication involving nonvertebrates have been reported in crustaceans, barley [20] and in the psychrophilic bacterium, *Bacillus psychrosaccharolyticus *[21]. In studies of crustacean LDH, protein electrophoresis has detected the presence of two *Ldh *loci in the northern krill *Meganyctiphanes norvegica*, the Antarctic krill *Euphausia superba *[22], the lobster *Homarus americanus *[23], the snow crab *Chionoecetes opilio *[24], the amphipod *Hyallela azteca *(J. Witt personal communication), the cladoceran *Daphnia magna *[25] and possibly *Daphnia carinata *[26] and *Daphnia cephalata *[27]. All of these crustaceans except *Daphnia *belong to the class, Malacostraca. Further work will be required to determine if a gene duplication occurred early in this crustacean lineage (as occurred in vertebrates), or if these duplicated genes have independent origins within the class. The two copies of *L-Ldh *in *D. pulex *group with one another in our phylogenetic analyses, suggesting that they do not represent an ancient crustacean duplication, but analysis of other branchiopods will be required to determine when this duplication occurred. Moreover, the MP and NJ trees suggest that insects and crustaceans are reciprocally monophyletic, but the crustaceans are paraphyletic relative to the insects in the BI tree, with strong support. The possibility of crustacean paraphyly has been suggested in other phylogenetic studies involving a variety of markers [28-30], but these relationships have not been definitively resolved.

The only other invertebrate in our analysis with multiple copies of *L-Ldh *is the leech, *H. robusta*, with three copies. Two of the copies appear to be unique to this taxon, but our sample of sequences is not sufficient to determine when this duplication occurred. A very long branch connects *Ldh-C *in *H. robusta *with the flatworm instead of the other annelids in the NJ tree, while *Ldh-B *clusters with the flatworm in the BI tree. Additional annelid and flatworm taxa must be analyzed to "break up" these branches, and to examine the relationship between these two phyla. What is clear from our phylogenetic analyses is that the LDH genes of Arthropods are significantly distinct from the LDH genes of other invertebrates, including annelid and nematode worms.

LDH allozyme polymorphism has been intensely studied in *Daphnia *including species with strong habitat specificity. For example,*D. pulex *inhabits mainly freshwater ponds throughout North America and Europe that lack fish, while its closest relative, *Daphnia pulicaria*, inhabits lakes and is able to coexist with fish, which are efficient predators of these limnetic zooplankters. The two ecological species can be distinguished based on a diagnostic LDH allozyme polymorphism with a "slow" (S) allele in *D. pulex *and a "fast" (F) allele in *D. pulicaria *[31]. However, the situation is complicated by hybridization and transitions from cyclical to obligate parthenogenesis [32,33]. Although there are distinct mitochondrial lineages in the two species in North America [34], there are many lake populations of *Daphnia *that have a *D. pulicaria *LDH profile (F), but a *D. pulex*-like mitochondrial DNA (T. Crease, unpublished data).

The maintenance of the LDH-F allele in lake populations of *Daphnia*, regardless of maternal origin, suggests that LDH genotypes differ in physiological performance, which may affect fitness. This is the case in the teleost, *Fundulus heteroclitus *[35], where the frequency of two LDH-B alleles shows clinal variation with latitude and environmental temperature. In addition, the fish allozymes show differences in kinetics related to temperature suggesting selection has favored a particular form of the LDH protein. Further studies on *Ldh *genes from populations of both the lake and pond *Daphnia *species are necessary to determine which of the two *L-Ldh *loci is responsible for the S/F polymorphism, and to provide evidence for the presence or absence of selection on the polymorphic locus. This work will be complemented by the examination of the enzyme kinetics of the allozymes themselves.

### D-lactate dehydrogenases

Enzymes that oxidize D-lactic acid have been mainly identified in lower organisms such as prokaryotes and fungi (e.g., *Lactobacillus*, *Escherichia coli*, yeast) in which they play an important role in anaerobic energy metabolism. While most invertebrates preferentially oxidize L-lactic acid, several species of mollusks (the oyster *Crassostrea virginica*, the mussel *Mytilus edulis*, the limpet *Acmaea unicolor*, the chiton *Middendorffia caprearum*, the octopus *Eledone cirrosa*), a few arthropods (horseshoe crab, spiders, scorpions) and one polychaete, *Nereis *sp., were found to have exclusively D-LDH enzymatic activity [36,37]. Therefore, it had been suggested that the L-LDH and D-LDH enzymes are mutually exclusive. However, Flick and Konieczny [5] identified and characterized a putative mammalian D-LDH enzyme that shows substantial similarity to three yeast proteins with D-LDH activity. This suggests that mammals have the two naturally occurring forms (D- and L-) of LDH. Moreover, we identified a *D-Ldh *gene in the genomes of several taxa that also possess *L-Ldh *including *D. pulex*, several non-mammalian vertebrates, the urchin, *S. purpuratus*, and *C. elegans*, suggesting that the possession of both forms of this enzyme is phylogenetically widespread. Further research will be required to determine the phylogenetic distribution of *D-Ldh *in animals, to understand why it has been retained in some groups but not others, and to determine the different roles that L-LDH and D-LDH play in such diverse animals. Comparisons of taxa with both types of enzymes to close relatives with only one type would be informative in this regard. In addition, it will be interesting to determine whether the "missing" enzyme in such cases is the result of gene inactivation, degradation into a pseudogene, deletion, or is expressed at such low levels that the enzyme is not detected in typical assays.

## Conclusion

The presence of both *L-Ldh *and *D-Ldh *in several chordates and invertebrates suggests that the two enzymatic forms are not necessarily mutually exclusive. Moreover, the high degree of sequence similarity among D-LDH amino acid sequences suggests that they share a common evolutionary history. No recent duplications of *D-Ldh *have yet been observed. In contrast, the *L-Ldh *gene family is characterized by a history of duplication and deletion events, particularly within vertebrates. However, duplications have also been identified in several invertebrate taxa suggesting that the occurrence of isozymes whose activity is specific to different tissues or developmental stages is a common theme in L-LDH evolution.

## Methods

### Alignments and phylogenetic analyses

LDH sequences from vertebrates, invertebrates and plants were obtained from the GenBank and Swiss-Prot/EMBL data bases (Table 1) using a combination of queries based on the term "Lactate dehydrogenase" and BLAST searches. In the latter case, several well described vertebrate and invertebrate sequences were used as the query. Blast searches were also performed on invertebrate genome projects from which a few unannotated sequences were retrieved. The deduced amino acid sequence for the porcelain crab, *Petrolisthes cinctipes *was obtained from a cloned EST that contained the full length cDNA sequence [38]. The alignment of amino acid sequences was conducted using CLUSTAL W [39] with a gap opening penalty of 10 and a gap length penalty of 0.1. Minor adjustments to the alignments were made manually. To avoid ambiguity due to extensive sequence variability and length variability in the amino terminal arm, alignment positions 1–30 were removed from the L-LDH alignment in all analyses. Without this ambiguous region, the alignment of the ingroup taxa included six gaps, while the addition of plant sequences to the alignment resulted in four additional gaps. Phylogenetic analyses were inferred in MEGA4 [40], PAUP, version 4.0 [41] and MrBayes V3.1.2. [42] using three analytical approaches: Neighbor-joining (NJ), Maximum parsimony (MP) and Bayesian inference (BI). NJ trees were constructed from pairwise amino acid distances estimated using a Poisson correction. MP trees were estimated using a heuristic search algorithm with 100 replicates, sequences added at random and tree bisection-reconnection branch swapping. Amino acids were treated as unordered characters with equal weight and gaps were treated as "missing". The stability of both phylogenetic hypotheses was assessed with bootstrap analyses (100 replicates for MP and 1000 replicates for NJ). Analysis of the amino acid data was also conducted using the Bayesian inference (BI) method with a fixed rate model of amino acid substitution. The fixed rate model WAG was estimated by allowing "model jumping" between nine fixed-rate amino acid models. Runs of 1,000,000 generations were executed, with a sampling frequency of 10, a burn-in parameter of 25,000. Stability of the likelihood scores was assessed in preliminary trials before setting the burn-in parameter. To confirm that the results converged to the same topology, we repeated the analysis three times.

**Table 1:** List of sequences included in the phylogenetic analyses.

Assignment	Organisms		Accession number	Reference
**L-LDH** **EC 1.1.1.27**				
LDH	*Aedes aegypti*	yellow fever mosquito	[Swiss-Prot:Q16ND1]	[43]
LDH-A	*Alligator mississippiensis*	American alligator	[Swiss-Prot:Q9PW06]	[13]
LDH-B	*Alligator mississippiensis*	American alligator	[Swiss-Prot:Q9PW05]	[13]
LDH	*Anopheles gambiae*	African malaria mosquito	[Swiss-Prot:Q7Q981]	[44]
LDH	*Arabidopsis thaliana*	mouse-ear cress	[Swiss-Prot:O49191]	Dolferus et al. 1998 unpublished
LDH	*Caenorhabditis briggsae*	nematode	[Swiss-Prot:Q61ZF2]	[45]
LDH	*Caenorhabditis elegans*	nematode	[Swiss-Prot:Q27888]	[46]
LDH	*Capitella capitata*	segmented worm	gi 134102	unpublished
LDH	*Carcinus maenas*	green crab	[Swiss-Prot:DY308423]	[47]
LDH-B	*Chelodina siebenrocki*	turtle	[Swiss-Prot:Q6S5M2]	Ho and Li 2003 unpublished
LDH	*Ciona intestinalis*	tunicate	gi 149293	unpublished
LDH-A	*Cyprinus carpio*	common carp	[Swiss-Prot:Q9W7K5]	Tsoi et al. 1998 unpublished
LDH	*Clonorchis sinensis*	flatworm	gi 56131044	unpublished
LDH-A	*Danio rerio*	zebrafish	[Swiss-Prot:Q9PVK5]	Tsoi et al. 1998 unpublished
LDH-B	*Danio rerio*	zebrafish	[Swiss-Prot:Q9PVK4]	Tsoi et al. 1998 unpublished
LDH-A	*Daphnia pulex*	water flea	gi 230172	unpublished
LDH-B	*Daphnia pulex*	water flea	gi 61140	unpublished
LDH	*Drosophila melanogaster*	fruit fly	[Swiss-Prot:Q95028]	[48]
LDH	*Drosophila pseudoobscura*	fruit fly	[Swiss-Prot:Q29FH9]	[49]
LDH-A	*Fundulus heteroclitus*	killifish	[Swiss-Prot:Q92055]	[50]
LDH-B	*Fundulus heteroclitus*	killifish	[Swiss-Prot:P20373]	[9]
LDH-C	*Fundulus heteroclitus*	killifish	[Swiss-Prot:Q06176]	[51]
LDH-A	*Gallus gallus*	chicken	[Swiss-Prot:P00340]	[52]
LDH-B	*Gallus gallus*	chicken	[Swiss-Prot:P00337]	Tsoi et al. 1998 unpublished
LDH-A	*Helobdella robusta*	segmented worm	gi 115469	unpublished
LDH-B	*Helobdella robusta*	segmented worm	gi 156879	unpublished
LDH-C	*Helobdella robusta*	segmented worm	gi 166102	unpublished
LDH-A	*Homo sapiens*	human	[Swiss-Prot:P00338]	[53]
LDH-B	*Homo sapiens*	human	[Swiss-Prot:P07195]	[54]
LDH-C	*Homo sapiens*	human	[Swiss-Prot:P07864]	[55]
LDH-A	*Hordeum vulgare*	barley	[Swiss-Prot:P22988]	[20]
LDH-B	*Hordeum vulgare*	barley	[Swiss-Prot:P22989]	[20]
LDH	*Litomosoides sigmodontis*	nematode	[GenBank:DN558179]	Gregory W. 1995 unpublished
LDH-A	*Monodelphis domestica*	gray short-tailed opossum	[Swiss-Prot:Q9XT87]	Tsoi et al. 1998 unpublished
LDH-B	*Monodelphis domestica*	gray short-tailed opossum	[Swiss-Prot:Q9XT86]	Tsoi et al. 1998 unpublished
LDH-A	*Mus musculus*	house mouse	[Swiss-Prot:P06151]	[56]
LDH-B	*Mus musculus*	house mouse	[Swiss-Prot:P16125]	[57]
LDH-C	*Mus musculus*	house mouse	[Swiss-Prot:P00342]	[58]
LDH	*Petrolisthes cinctipes*	flat porcelain crab	[GenBank:FE768558FE768559]	[35]
LDH	*Petromyzon marinus*	sea lamprey	[Swiss-Prot:P33571]	[10]
LDH-A	*Squalus acanthias*	spiny dogfish	[Swiss-Prot:P00341]	[59]
LDH-B	*Squalus acanthias*	spiny dogfish	[Swiss-Prot:Q9YI05]	[60]
LDH	*Strongylocentrotus purpuratus*	purple urchin	[GenBank:XP_001196488]	unpublished
LDH	*Styela plicata*	tunicate	[Swiss-Prot:O44340]	[7]
LDH	*Tribolium castaneum*	red flour beetle	[GeneBank:XM_963110]	unpublished
LDH-A	*Xenopus laevis*	African clawed frog	[Swiss-Prot:P42120]	[11]
LDH-B	*Xenopus laevis*	African clawed frog	[Swiss-Prot:P42119]	[11]
LDH-C	*Xenopus laevis*	African clawed frog	[Swiss-Prot:P42121]	[11]
LDH	*Zea mays*	maize	[Swiss-Prot:P29038]	[61]
**D-LDH EC** **1.1.2.4**				
	*Arabidopsis thaliana*	mouse-ear cress	[GeneBank:NM_120741]	unpublished
	*Bos taurus*	cattle	[Swiss-Prot:Q148K4]	Moore et al. 2006 unpublished
	*Caenorhabditis elegans*	nematode	[GeneBank:NP_001023872]	unpublished
DLD1	*Candida sphaerica*	yeast	[Swiss-Prot:Q12627]	[62]
	*Canis familiaris*	dog	[GeneBank:XP_852976]	unpublished
	*Ciona intestinalis*	tunicate	gi: 289925	unpublished
	*Capitella capitata*	segmented worm	gi: 177524	unpublished
	*Danio rerio*	zebrafish	[GeneBank:NP_956157]	[63]
	*Daphnia pulex*	water flea	gi: 302238	unpublished
	*Lottia gigantea*	gastropod	gi: 229861	unpublished
	*Helobdella robusta*	segmented worm	gi: 185793	unpublished
	*Homo sapiens*	human	[Swiss-Prot:Q86WU2]	[5]
	*Monodelphis domestica*	gray short-tailed opossum	[GeneBank:XP_001375142]	unpublished
	*Mus musculus*	house mouse	[Swiss-Prot:Q7TNG8]	[5]
	*Nematostella vectensis*	starlet sea anemone	[GeneBank:XP_001626025]	unpublished
	*Pan troglodytes*	chimpanzee	[GeneBank:XP_001139010]	unpublished
	*Rattus norvegicus*	Norway rat	[Swiss-Prot:Q7TPJ4]	Xu et al. 2003 unpublished
DLD1	*Saccharomyces cerevisiae*	baker's yeast	[Swiss-Prot:YDL174C]	[64]
	*Strongylocentrotus purpuratus*	purple urchin	[GeneBank:XP_796456]	unpublished
	*Tetraodon nigroviridis*	Green puffer	[Swiss-Prot:Q4T6R2]	[65]
	*Xenopus laevis*	African clawed frog	[Swiss-Prot:A1L2R0]	[66]

## Authors' contributions

The data were collected and analyzed by MEC and TJC. The manuscript was drafted by MEC and TJC with contributions by DJI and JHS. All authors participated in the project design, and read and approved the final manuscript.
